# Prognostic Significance of Gene Expression and DNA Methylation Markers in Circulating Tumor Cells and Paired Plasma Derived Exosomes in Metastatic Castration Resistant Prostate Cancer

**DOI:** 10.3390/cancers13040780

**Published:** 2021-02-13

**Authors:** Martha Zavridou, Areti Strati, Evangelos Bournakis, Stavroula Smilkou, Victoria Tserpeli, Evi Lianidou

**Affiliations:** 1 Analysis of Circulating Tumor Cells Laboratory, Lab of Analytical Chemistry, Department of Chemistry, National and Kapodistrian University of Athens, 15771 Athens, Greece; mzavridou@chem.uoa.gr (M.Z.); astrati@chem.uoa.gr (A.S.); ssmilkou@chem.uoa.gr (S.S.); victserp@chem.uoa.gr (V.T.); 2 Oncology Unit, 2nd Department of Surgery, Aretaieio Hospital, Medical School, National and Kapodistrian University of Athens, 11528 Athens, Greece; vagimith@yahoo.com

**Keywords:** prostate cancer, liquid biopsy, CTCs, extracellular vesicles, exosomes, DNA methylation, gene expression, RT-qPCR, MSP

## Abstract

**Simple Summary:**

“Liquid biopsy”, based on the analysis of circulating tumor cells (CTCs) and circulating tumor DNA (ctDNA), provides non-invasive real-time monitoring of tumor evolution and therapeutic efficacy. We performed for the first time a direct comparison study on gene expression and DNA methylation markers in CTCs and paired plasma-derived exosomes and evaluated their prognostic significance in metastatic castration resistant prostate cancer. Our results revealed for the first time a significantly higher positivity of all markers in EpCAM-positive CTCs compared to plasma-derived exosomes. We report that in EpCAM-positive CTCs, *CK-19*, *PSMA*, *TWIST1* expression and *GSTP1* methylation are significantly correlated with worse overall survival (OS), while in exosomes, *CK-8* expression and *GSTP1* and *RASSF1A* methylation status were significantly correlated with a lower OS. We also enumerated CTC and tumor-derived extracellular vesicles (tdEVs) using CellSearch (CS) and found a correlation between the CTC and tumor-derived extracellular vesicles (tdEVs) enumeration values.

**Abstract:**

Liquid biopsy, based on the analysis of circulating tumor cells (CTCs) and circulating tumor DNA (ctDNA), provides non-invasive real-time monitoring of tumor evolution and therapeutic efficacy. We performed for the first time a direct comparison study on gene expression and DNA methylation markers in CTCs and paired plasma-derived exosomes and evaluated their prognostic significance in metastatic castration resistant prostate cancer. This prospective liquid biopsy (LB) study was based on a group of 62 metastatic castration resistant prostate cancer (mCRPC) patients and 10 healthy donors (HD) as controls. Identical blood draws were used to: (a) enumerate CTC and tumor-derived extracellular vesicles (tdEVs) using CellSearch (CS) and (b) analyze CTCs and paired plasma-derived exosomes at the gene expression and DNA methylation level. CTCs were enumerated using CellSearch in 57/62 patients, with values ranging from 5 to 854 cells/7.5 mL PB. Our results revealed for the first time a significantly higher positivity of gene expression markers (*CK-8*, *CK-18*, *TWIST1*, *PSMA*, *AR-FL*, *AR-V7*, *AR-567* and *PD-L1* mRNA) in EpCAM-positive CTCs compared to plasma-derived exosomes. *GSTP1*, *RASSF1A* and *SCHLAFEN* were methylated both in CTC and exosomes. In CTCs, Kaplan–Meier analysis revealed that *CK-19* (*p* = 0.009), *PSMA* (*p* = 0.001), *TWIST1* (*p* = 0.001) expression and *GSTP1* (*p* = 0.001) methylation were correlated with OS, while in exosomes *GSTP1* (*p* = 0.007) and *RASSF1A* (*p* = 0.001) methylation was correlated with OS. Our direct comparison study of CTCs and exosomes at gene expression and DNA methylation level, revealed for the first time a significantly higher positivity in EpCAM-positive CTCs compared to plasma-derived exosomes. Future perspective of this study should be the evaluation of clinical utility of molecular biomarkers in CTCs and exosomes on independent multicentric cohorts with mCRPC patients.

## 1. Introduction

Prostate cancer is the second most common cause of cancer-related death in men. Despite advances in screening, surgery, hormone therapy and chemotherapy, ~27,000 men still die in the USA each year from metastatic prostate cancer [1]. Nowadays, research in the field of diagnosis and treatment of prostate cancer focuses on finding biomarkers in blood. Liquid biopsy, based on the analysis of circulating tumor cells (CTCs), circulating tumor DNA (ctDNA), circulating miRNAs and extracellular vesicles (EVs) [2,3] provides a non-invasive, real-time monitoring of tumor evolution and therapeutic efficacy [4,5]. Prostate-specific antigen (PSA) and other standard markers are not ideal [6], as they still lack the required diagnostic specificity and prognostic value and they also have a high percentage of false positive results. Moreover, detection of early-stage prostate cancer is a major challenge for liquid biopsies [2,7].

CTCs are exceptionally heterogeneous, rare and show genetic differences from primary tumor cells. These differences alter the molecular balances responsible for cell adhesion, migratory capacity and angiogenesis and as a result CTCs form secondary tumors in distant organs [8]. Epithelial Mesenchymal Transition (EMT) plays a critical role in metastasis formation in all types of solid cancers; EMT results in down-regulation of epithelial markers like EpCAM and simultaneous up-regulation of certain mesenchymal proteins, and has been shown in CTCs [9]. CTC molecular characterization offers the unique potential to understand better the biology of metastasis and resistance to established therapies. CTC enumeration in peripheral blood, based on the CellSearch^®^ system (Menarini, Italy), is still the only FDA-cleared assay for patients with metastatic prostate cancer [10]. The robust and semi-automated CellSearch platform enriches CTCs based on EpCAM expression and identifies these cells based on the absence of CD45 and the presence of cytokeratin (*CK-8, CK-18, CK-19*) expression.

Exosomes, a subcategory of EVs secreted by living cells in the extracellular space or in blood circulation, are a valuable source proteins and lipids biomarkers [11]. The unique biogenesis of exosomes, their ubiquitous production by all cell types, and their biological features in liquid biopsies have generated excitement for their potential as a source of cancer biomarkers [11]. In cancer patients, exosomes are secreted by tumor cells and it is now believed that they hold an important role in the metastatic process [12,13]. Similar to CTCs, exosomes can be a source of quantitative and qualitative information.

Molecular assays for studying gene expression in CTCs and exosomes take advantage of the high sensitivity and specificity of RT-qPCR and can be used downstream to many different CTCs isolation systems. The detection of biomarkers on CTCs and exosomes is highly important for therapeutic decisions especially if these are indicative of response to specific treatments. Nowadays, detection of AR-V7 in CTCs can guide clinicians to select hormonotherapy or chemotherapy in mCRPC [14,15]. Moreover, detection of DNA methylation-based markers on CTCs and exosomes can provide important information on epigenetic silencing of genes that play a critical role in the biology of metastasis [16].

In this study we performed for the first time a direct comparison study of gene expression (*CK-19*, *CK-8*, *CK-18*, *TWIST1*, *ALDH1*, *PSMA*, *AR-FL*, *AR-V7*, *AR-567* and *PD-L1*) and DNA methylation (*GSTP1* and *RASSF1A*) markers in CTCs and paired plasma-derived exosomes and evaluated their prognostic significance in metastatic castration resistant prostate cancer.

## 2. Materials and Methods

**Figure 1:**
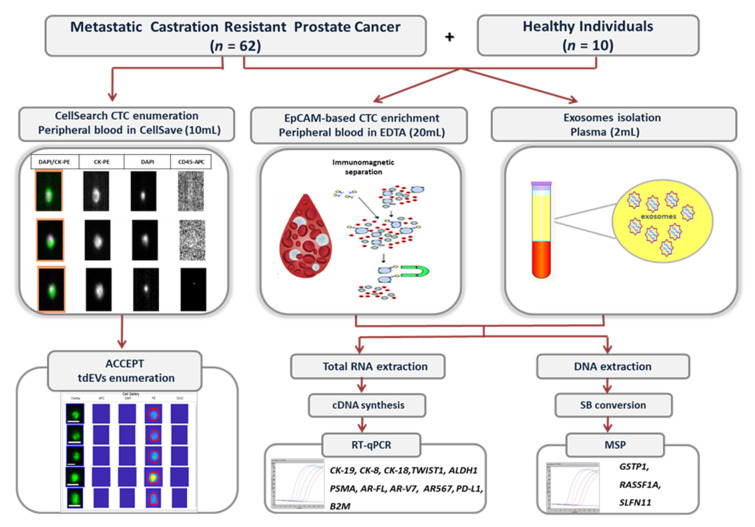
Experimental flowchart of the study.

The experimental flowchart is shown in Figure 1.

### 2.1. Peripheral Blood Samples Collection

Peripheral blood (PB) was prospectively collected from 62 mCRPC patients and 10 HD (male, age 30–60 years old). All mCRPC patients received chemotherapy or new hormonal agents (NHAs) prior to analysis. All patients gave a written informed consent to participate in the study, which was approved by the Ethics and Scientific Committee of Aretaieio University Hospital and their clinical characteristics at the time of diagnosis are presented in Table S1. The first 5 mL of blood were discarded to avoid contamination from skin epithelial cells [16]. PB was drawn into K2EDTA tubes (20 mL) (BD Vacutainer, Plymouth, UK) and Cellsave tubes (Menarini Silicon Biosystems) (10 mL). Blood samples were mixed immediately after the draw by inverting gently 10 times, and then were maintained at room temperature (RT). The same blood tubes and the same blood draws were used for both CTC and exosome analysis. All samples were processed for CTC and plasma isolation within 2 h.

### 2.2. Isolation of EpCAM-Positive CTCs and Exosomes

Capture beads, coated with the monoclonal antibody BerEP4 against the human epithelial antigen, EpCAM, were used for CTCs enrichment (Dynabeads^®^ Epithelial Enrich, Life Technologies, Waltham, MA, USA) as previously described [17,18]. Exosomes were isolated from 2 mL of plasma by affinity-based binding to a spin column (exoRNeasy Maxi kit, QIAGEN^®^, Hilden, Germany) as previously described [19]. Using this method, all exosomes were isolated.

### 2.3. RNA Extraction and cDNA Synthesis

Total RNA from CTCs was isolated using TRIZOL-LS (ThermoFisher Scientific, Waltham, MA, USA) as previously described [17]. RNA from exosomes was isolated by adding QIAzol to the column and the lysate was collected by centrifugation. Total RNA was isolated using exoRNeasy Maxi kit (QIAGEN^®^, Hilden, Germany). Isolated total RNA was dissolved in 14 μL of nuclease free water. cDNA synthesis was performed using the High-Capacity RNA-to-cDNA kit (Applied Biosystems, Waltham, MA, USA). Total RNA (100 ng/μL) isolated from MCF-7 cells was used as a positive control for cDNA synthesis [17].

### 2.4. RT-qPCR

For gene expression analysis, we applied our previously developed and analytically validated RT-qPCR assays for cytokeratins (*CK-19*, *CK-8* and *CK-18*), *TWIST* family transcription factor 1 (TWIST1), aldehyde dehydrogenase 1 (*ALDH1*), prostate-specific membrane antigen (*PSMA*), programmed death-ligand 1 (*PD-L1*) and beta-2-microglobulin (*Β2Μ*) (used as a reference gene). All RT-qPCR reactions were performed in the LightCycler^®^ 2.0 (IVD instrument, Roche Diagnostics, Mannheim, Germany) and in the Cobas z480 (Roche Diagnostics) following the MIQE guidelines [20]. The expression levels of *TWIST1*, *ALDH1* and *PD-L1* were normalized using the 2^−ΔΔCt^ approach in respect to the expression of Β2Μ. We additionally used our recently developed multiplex RT-qPCR for the simultaneous quantification of AR-full length (*AR-FL*) and *AR-V7*, *AR-567* splice variants using hydrolysis probes [19].

### 2.5. DNA Isolation

CTCs: gDNA was extracted from CTCs using the TRIZOL-LS reagent (ThermoFisher Scientific, Waltham, MA, USA) as previously described [21]. Isolated gDNA was dissolved in 30 μL of 8 mmol/L NaOH. Exosomes: gDNA was extracted from exosomes using QIAzol reagent (QIAGEN^®^, Hilden, Germany). Isolated gDNA was dissolved in 30 μL of 8 mmol/L NaOH.

### 2.6. Sodium Bisulfite (SB) Treatment

gDNA samples were treated with SB, to convert all non-methylated cytosines to uracil, while methylated cytosines were not converted, using the EZ DNA Methylation Gold Kit (ZYMO Research, Irvine, CA, USA) as previously described [21]. SB-treated DNA was stored at −70 °C until use. In each SB reaction, dH_2_O and DNA were included as negative and positive control, respectively.

### 2.7. Real-Time Methylation Specific PCR (Real-Time MSP)

We used our previously developed and validated highly specific and sensitive real-time MSP assays for *GSTP1* and *RASSF1A*. We developed a novel real-time MSP assay for *SCHLAFEN* (*SLFN11*). The quality of SB-converted DNA was first checked by real-time MSP for β-actin (*ACTB*). Samples in which *ACTB* was not amplified and as a result no SB-converted DNA was detected were excluded from the study. All experiments for *GSTP1*, *RASSF1A* [22] and *SLFN11* methylation analyses were performed in the 96-well plates of Cobas z480 system in a total volume of 10 μL (1 μL of SB-converted DNA was added to 9 μL reaction mixture). Universal Methylated Human DNA Standard (ZYMO Research, Irvine, CA, USA) was used as fully methylated (100%) positive control. The MSP assays for *GSTP1* and *RASSF1A* are not quantitative, so we report a sample as methylation positive, when we detect an MSP amplification signal Cq < 40.00 and as methylation negative only in the total absence of amplification signal.

### 2.8. CTCs and Tumor-Derived EVs (tdEVs) Enumeration

CellSearch^®^ was used for the enumeration of CTCs in 57/62 patients according to manufacturer’s instructions. We could not perform CS analysis for 5 samples because no additional peripheral blood was sent in the lab in CellSave tubes. For tdEVs enumeration, the digitally stored fluorescence image files were reanalyzed with the open-source ACCEPT software v1.1 (http://github.com/LeonieZ/ACCEPT) using the “Full Detection” function [23].

### 2.9. Statistical Analysis

All statistical analysis was performed by using the SPSS version 25.0 (IBM^®^ SPSS^®^ Statistics). The Kaplan–Meier method was used for the calculation of overall survival (OS) curves and log-rank test was performed for the comparisons Cox proportional hazards (PH) models were used to evaluate the relationship between molecular markers and event-time distributions, with CTC count and Gleason score. *p*-values <0.05 were considered as statistically significant.

## 3. Results

### 3.1. Gene Expression Analysis

**Figure 2:**
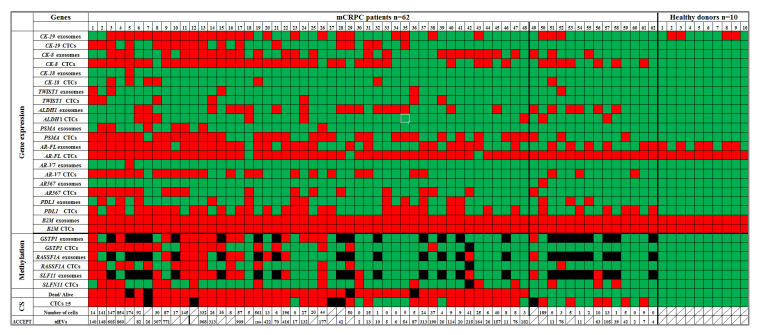
Heatmap: Direct comparison of gene expression and DNA methylation markers in circulating tumor cells (CTCs) and paired plasma-derived exosomes in mCRPC patients and HD.

EpCAM-positive CTCs: All samples were of excellent RNA quality as this was certified by RT-qPCR for Β2Μ. All 10 HD samples were negative for CK-19, CK-8 and CK-18 expression. In mCRPC, CK-19 was detected in 18/62 (29.0%), CK-8 in 41/62 (66.1%) and CK-18 in 7/62 (11.3%) samples. Relative fold change values (2^−ΔΔCt^) for TWIST1, ALDH1 and PD-L1 were normalized according to B2M; TWIST1 was overexpressed in 7/62 (11.3%), ALDH1 in 8/62 (12.9%) and PD-L1 in 34/62 (54.8%) samples. In HD, AR-FL was expressed in 10/10 (100%) as expected while all 10 HD samples were negative for AR-V7 and AR-567 splice variants. In mCRPC, AR-FL transcripts were detected in 59/62 (95.2%), AR-V7 in 25/62 (40.3%) and AR-567 in 18/62 (29.0%) samples. Finally, PSMA was detected in 35/62 (56.5%) samples (Figure 2). Based on the gene expression analysis, samples CK-positive and/or PSMA positive were considered as CTC positive. Our CTC-genes signature revealed that 49/57 (85.9%) samples were defined as CTC-positive. According to CS, 49/57 (85.9%) were found to have at least one CTC. The concordance rate between CTC-genes signature on EpCAM(+) CTC fractions and CellSearch was 45/57 (78.9%).

Exosomes: All cDNA samples derived from exosomes from paired plasma (2 mL) were of excellent RNA quality as this was certified by RT-qPCR for *B2M*. *CK-19* was not a specific marker in exosomes of mCRPC patients since in the group of HD, *CK-19* expression was detected in 4/10 (40.0%) cases. In mCRPC samples, *CK-19* was detected in 28/62 (45.2%). All 10 HD samples were negative for *CK-8* and *CK-18* expression. In mCRPC samples, *CK-8* was detected in 27/62(43.5%) and *CK-18* in 1/62(1.6%). Relative fold change values (2^−ΔΔCt^) for *TWIST1*, *ALDH1* and *PD-L1* were normalized according to *B2M*. *TWIST1* was overexpressed in 5/62(8.0%) samples, *ALDH1* in 23/62(37.1%) and *PD-L1* in 15/62 (24.2%). The simultaneous quantification of *AR-FL* and *AR-V7* and *AR-567* splice variants has shown that in the group of HD, all samples were negative for *AR-V7* and *AR-567* splice variants while *AR-FL* was expressed in 6/10 (60%) cases as expected. In mCRPC samples, *AR-FL* transcripts were detected in 37/62 (59.7%), *AR-V7* in 1/62(1.6%) and *AR-567* in 1/62 (1.6%) samples. *PSMA* was detected in 7/62 (11.3%) samples (Figure 2).

### 3.2. DNA Methylation

EpCAM-positive CTCs: All 10 HD plasma samples processed exactly in the same way as the mCRPC samples, were negative for *GSTP1*, *RASSF1A* and *SLFN11* methylation (0/10, 0%). In mCRPC, *GSTP1* methylation was detected in 18/61(29.5%), *RASSF1A* methylation in 14/61(23.0%) and *SLFN11* methylation 15/61(24.6%). Exosomes: In exosomes, all 10 HD plasma samples, processed exactly in the same way as the mCRPC samples, were negative for *GSTP1*, *RASSF1A* and *SLFN11* methylation (0/10, 0%. 38/62 SB-converted DNA samples passed the quality control and were further analyzed for DNA methylation. In mCRPC, *GSTP1* methylation was detected in 14/38(36.8%), *RASSF1A* in 11/38 (28.9%) and *SLFN11* 15/38(39.5%) samples.

### 3.3. Direct Comparison of Gene Expression Markers between Plasma-Derived Exosomes and Paired EpCAM-Positive CTCs

The concordances between EpCAM-positive CTCs versus exosomes for each gene of interest were estimated using the χ2 test for 62 mCRPC paired samples. We did not observe any statistically significant correlation in these samples for any of the genes tested (Table S2). A higher percentage of *CK-8*, *CK-18*, *TWIST1*, *PSMA*, *AR-FL*, *AR-V7*, *AR-567* and *PD-L1* positive samples was found in EpCAM-positive CTCs in comparison to plasma-derived exosomes (Figure S1). *CK-19* was excluded from this comparison, since it was not specific in exosomes. In EpCAM-positive CTCs, 43/62 (69.4%) mCRPC patients were positive in at least one *CK* (*CK-19*, *CK-8*, *CK-18*). In exosomes, 28/62 (45.2%) mCRPC patients were positive in at least one *CK* (*CK-8*, *CK-18*). Only *ALDH1* was expressed at higher levels in exosomes than in EpCAM-positive CTCs.

The concordances between EpCAM-positive CTCs versus exosomes for DNA methylation of *GSTP1* and *RASSF1A* between were estimated using the χ2 test for 38 mCRPC paired samples. We observed statistically significant correlation for the above samples both for *GSTP1* (*p* = 0.021) and *RASSF1A* (*p* = 0.019) (Table S2). The concordance between DNA methylation on EpCAM-positive CTCs and paired exosomes was (74%) for *GSTP1*, (79%) for *RASSF1A* and (66%) for *SLFN11* (Table S2). A higher percentage of *GSTP1*, *RASSF1A* and *SLFN11* positive samples was found in plasma-derived exosomes in comparison to EpCAM-positive CTCs (Figure S1).

### 3.4. Survival Analysis

**Figure 3:**
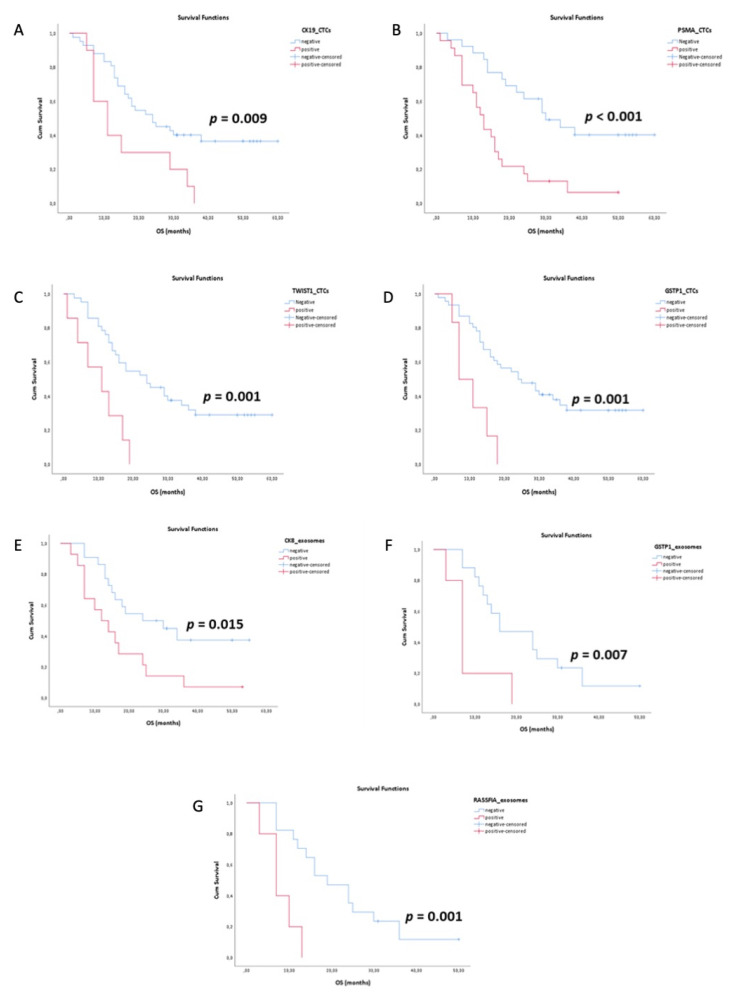
Kaplan–Meier estimates of overall survival (OS) in CTCs: (**A**) CK-19, (**B**) PSMA, (**C**) TWIST1, (**D**) GSTP1 and in exosomes (**E**) CK-8, (**F**) GSTP1, (**G**) RASSF1A.

Kaplan-Meier analysis was performed to estimate the correlation between OS with gene expression and DNA methylation markers. In EpCAM-positive CTCs, the survival curves for *CK-19*, *PSMA* and *TWIST1* expression were significantly correlated with OS. Patients positive for *CK-19* had significantly different OS (16.2 mo vs. 32.4 mo, *p* = 0.009, long-rank test, (Figure 3A) compared to negative patients. Patients positive for *PSMA* and *TWIST1* had significantly different OS (16.0 mo vs. 36.5 mo, *p* < 0.001, long-rank test, (Figure 3B) and 10.2 mo vs. 30.1 mo, *p* = 0.001, long-rank test, Figure 3C) compared to negative patients. *GSTP1* methylation was significantly correlated with a lower OS (10.5 mo vs. 31.3 mo, *p* = 0.001, long-rank test, Figure 3D). In plasma-derived exosomes, *CK-8* expression was significantly correlated with OS (16.9 mo vs. 31.8 mo, *p* = 0.015, long-rank test, Figure 3E). *GSTP1* and *RASSF1A* methylation was significantly correlated with a lower OS (8.6 mo vs. 21.4 mo, *p* = 0.007 and 8.0 mo vs. 22.6 mo, *p* = 0.001, long-rank test, Figure 3F,G).

**Figure 4:**
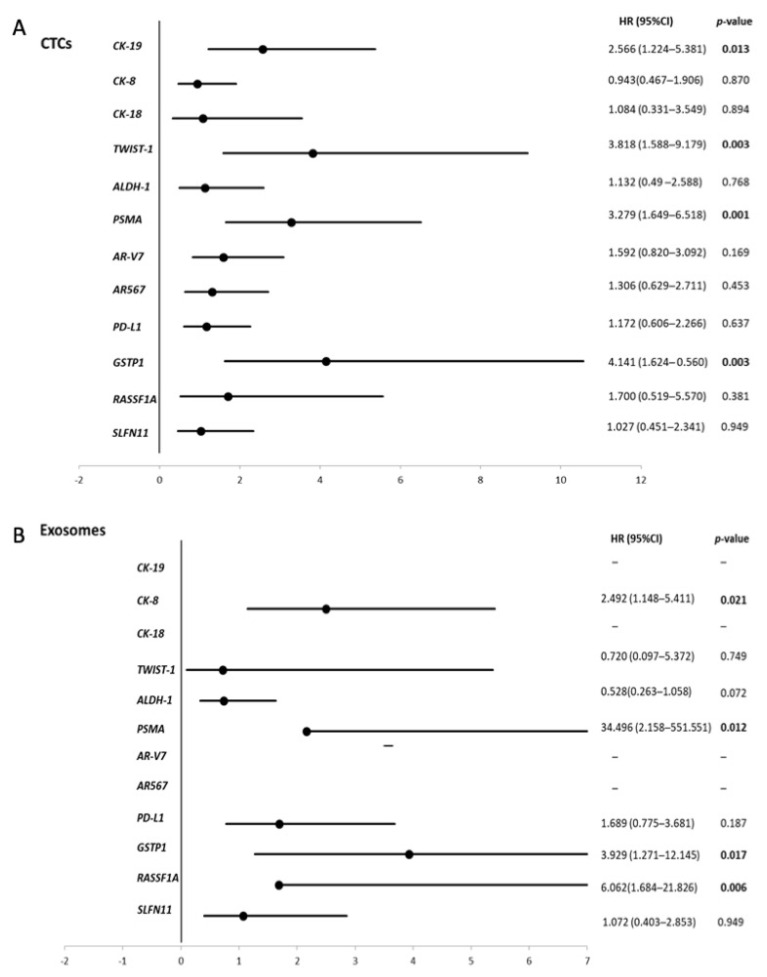
Forest plots of univariate Cox models for OS of mCRPC patients: (**A**) CTCs and (**B**) exosomes. Bold values denote statistical significance at *p* < 0.05.

In CTCs, univariate analysis showed a significantly higher risk of death in the group of patients positive for *CK-19* (HR: 2.566, *p* = 0.013), *TWIST1* (HR: 3.818, *p* = 0.003), *PSMA* (HR: 3.279, *p* = 0.001) and *GSTP1* methylation (HR: 4.141, *p* = 0.003) (Figure 4A). In plasma-derived exosomes, univariate analysis showed a significantly higher risk of death in the group of patients positive for *CK-8* expression (HR: 2.492, *p* = 0.021), *GSTP1* methylation (HR: 3.929, *p* = 0.017) and *RASSF1A* methylation (HR: 6.162, *p* = 0.006) (Figure 4B).

In CTCs, multivariate analysis confirmed the prognostic significance of individual *CK-19* (HR:2.609, *p* = 0.018), *TWIST1* (HR: 4.421, *p* = 0.002), *PSMA* (HR:3.242, *p* = 0.002) and *GSTP1* methylation (HR:3.111, *p* = 0.024) independently from patients’ CTC count and Gleason score. Combining all molecular markers in a single multivariate analysis (*CK-19*, *TWIST1*, *PSMA* and *GST1* methylation) *TWIST1*, *PSMA* provided the strongest increment of death risk (Table 1).

### 3.5. CTCs and tdEVs Enumeration

**Figure 5:**
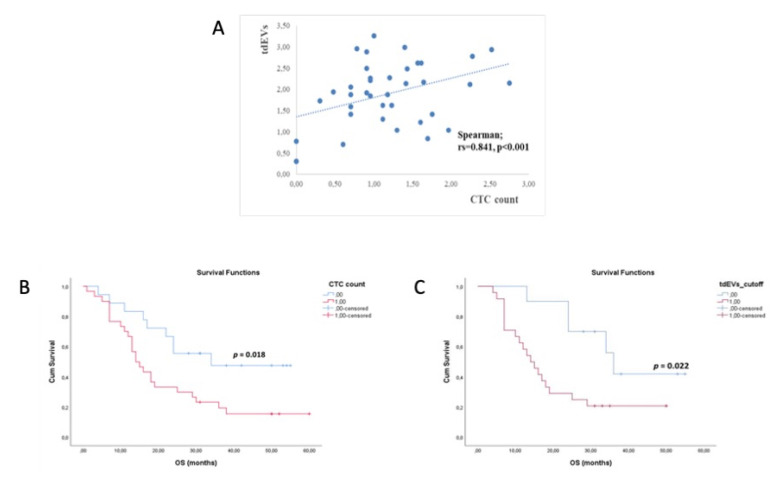
**(A**) Spearman correlation between CTC counts and tumor-derived extracellular vesicle (tdEV) numbers, (**B**) Kaplan–Meier estimates of OS for CTC count, (**C**) Kaplan–Meier estimates of OS for tdEVs (cutoff value 20 tdEVs/7.5 mL PB.

Using the CellSearch^®^ system more than ≥5 CTC/7.5 mL were detected in 42/57 (73.7%) patient samples (Figure 2). CTCs counts ranged from 5 to 854 cells/7.5 mL PB. 46/62 samples were evaluable for automated enumeration of tdEVs. Based on the study by Nanou et al. [22], the range of tdEVs in HD is 0–20 per 7.5 mL PB so we used as cutoff value 20 tdEVs in our study. 38/46 samples had ≥20/7.5 mL PB of tdEVs (median:144, range: 20–1804). We report a strong positive correlation between CTC counts and tdEVs counts (Spearman correlation coefficient rs = 0.841, *p* < 0.001) (Figure 5A), and the concordance was 38/46 (82.6%) (Chi-squared; *p* < 0.001). More specifically, CTCs (≥5 cells/7.5 mL PB) and tdEVs (≥20/7.5 mL PB) were detected in 29/46 samples (63.0%). CTCs (<5 cells/7.5 mL PB) and tdEVs (<20/7.5 mL PB) were detected in 9/46 (19.6%). The survival curve for CTCs (5 cells/7.5 mL) was significantly correlated with OS compared to patients with 5 cells/7.5 mL (22.4mo vs. 35.8mo, *p* = 0.018, long-rank test, (Figure 5B). Patients with tdEVs 20/7.5 mL had significantly different OS (20.5mo vs. 39.0mo, *p* = 0.022, long-rank test, (Figure 5C). The survival curve for tdEVs was significantly correlated with OS. Patients with tdEVs 20/7.5 mL had significantly different OS (20.5mo vs. 39.0mo, *p* = 0.022, long-rank test, (Figure 5C)) compared to patients with tdEVs 20/7.5 mL PB.

## 4. Discussion

We report for the first time results on a direct comparison of gene expression and DNA methylation markers in CTCs and paired plasma-derived exosomes and their prognostic significance in metastatic castration resistant prostate cancer. Current diagnostic assays for prostate cancer, including serum PSA, lack sufficient specificity and sensitivity to determine the aggressiveness of the disease and to identify appropriate treatment [24]. Additional reliable biomarkers are needed to facilitate early diagnosis of prostate cancer, determine the patient’s prognosis and predict responses to a given therapeutic intervention [2,25]. 

Several groups including ours have verified the importance of using molecular assays for CTC molecular characterization [7,16,17,18,19,23,26]. We have already shown that molecular assays based on real-time PCR carried out in nucleic acids material (RNA or genomic DNA) isolated from the EpCAM-positive CTC fraction can give valuable information for the molecular characterization of CTC at the gene expression, DNA methylation and DNA mutation level [4,16].

In the present study, we compared directly in the same blood draws the expression of *CK-19*, *CK-8*, *CK-18*, *TWIST1*, *ALDH1*, *PSMA*, *AR-FL*, *AR-V7*, *AR-567es*, *PD-L1* and three DNA methylation markers (*GSTP1*, *RASSF1A* and *SLFN11*) in CTCs and paired plasma-derived exosomes. Our results reveal a remarkable heterogeneity on gene expression and DNA methylation markers in EpCAM-positive CTCs and paired exosomes in mCRPC patients.

Different CKs including *CK8*, *CK18* and *CK19*, as the most abundant CKs of epithelial cells, are detected in cancers. According to our results, *CK-19* expression was detected in EpCAM-positive CTCs of mCRPC patients and correlated with OS. In plasma-derived exosomes, *CK-19* expression was detected in 4/10 (40.0%) cases of HD and in 28/62 (45.2%) cases of mCRPC patients. Hence, *CK-19* expression was not specific in exosomes. In exosomes, *CK-8* and *CK-18* were not detected in HD and detected in mCRPC but in lower percentages when compared to CTCs. *CK-8* expression in plasma-derived exosomes was significantly correlated with OS. Our comparison study revealed also that *TWIST1* and *ALDH1* were highly expressed in EpCAM-positive CTCs and paired plasma-derived exosomes. In EpCAM-positive CTCs, *TWIST1* was correlated with OS.

According to our results, *PSMA* expression was detected both in CTCs and exosomes. Gorges et al. reported a high level of intra patient heterogeneity in *PSMA* expression on CTCs, as well as discrepancies between *PSMA* protein expression in primary tumor tissue and corresponding CTCs [27]. Relative changes in *PSMA* expression on CTCs could also serve as a biomarker of AR signaling to evaluate AR activity in patients with CRPC [27]. Our results have shown that *PSMA* expression in EpCAM-positive CTCs, was significantly correlated with lower OS. However, in a recent study Kessel et al. have shown that *PSMA* expression does not display strong prognostic ability [28]. This could be due to different isolation and detection methods of CTCs.

The presence of *AR-V7* in CTCs was shown to predict resistance to new generation anti-AR-targeted treatments (abiraterone and enzalutamide) but not to taxane-based chemotherapy in mCRPC [29,30,31]. The development of molecular assays for the detection of *AR-V7* with high analytical sensitivity, specificity and accuracy is very important for the application of this biomarker in clinical practice [19,32,33]. Sharp et al. recently showed that patients who have no detectable CTCs by AdnaTest frequently have CTCs by CellSearch and express AR-V7 protein in matched tumor tissue [34]. We observed a higher positivity ratio of *AR-V7* expression in EpCAM-positive CTCs when compared directly to plasma-derived exosomes. Our results are in accordance with those reported by Nimir et al., who after a direct comparison in 16 samples, reported that *AR-V7* detection from CTCs showed higher sensitivity and have proven specificity compared to the detection from ctRNA and exosomes [35].

*PD-L1* expression consists a valuable prognostic and predictive biomarker for *PD-1* inhibitor sensitivity in a variety of cancers. Our group has previously developed a highly sensitive, specific and robust RT-qPCR assay of *PD-L1* mRNA expression and we reported its clinical utility in EpCAM-positive CTCs of head and neck squamous cell carcinoma patients [36]. In prostate cancer, the administration of immunotherapy against PD-L1 positive mCRPC patients proves antitumor activity [37]. It has been reported that *PD-L1* expression in exosomes represents a suitable immunotherapy target [38]. Our results clearly indicate *PD-L1* overexpression both in EpCAM-positive CTCs and paired plasma-derived exosomes. A higher percentage of *PD-L1* positive samples was found in EpCAM-positive CTCs in comparison to plasma-derived exosomes, and this could possibly be explained by the fact that used different amounts of sample for the analysis; exosomes were isolated from 2 mL of plasma while CTCs were isolated from 20 mL of PB.

Analysis of DNA methylation in CTCs can give important information on the molecular and biological nature of these cells. In this study we directly compared for the first time *GSTP1* and *RASSF1A* methylation in EpCAM-positive CTCs and exosomes in identical blood draws. Changes in GSTP1 activity and expression have been reported in many tumors and this is largely due to the DNA hypermethylation of *GSTP1*. Our results clearly indicate that GSTP1 and *RASSF1A* were highly methylated both in EpCAM-positive CTCs and paired plasma-derived exosomes. Moreover, *GSTP1* methylation significantly correlates with a low OS in EpCAM-positive CTCs and paired plasma-derived exosomes, while RASSF1A methylation was significantly correlated with OS in plasma-derived exosomes. This is the first study on the evaluation of *GSTP1* and *RASSF1A* methylation in EpCAM-positive CTCs and paired plasma-derived exosomes of mCRPC patients.

*SLFN11* is ubiquitously expressed in the human body. High expression has been associated with response to DDAs while, on the other hand, low expression has been implicated in resistance to therapy and subsequently poor prognosis in a variety of cancers [39,40,41,42]. However, only in a small amount of studies, this low expression of *SLFN11* in primary cancers as well as in commonly used cancer cell lines has been associated with epigenetic silencing, and mainly, DNA hypermethylation of the promoter [42]. In this study we developed a real-time MSP assay for *SLFN11*. Our results clearly indicate that *SLFN11* was methylated both in EpCAM-positive CTCs and paired plasma-derived exosomes.

The prognostic significance of *CK-19*, *TWIST1*, *PSMA* expression and *GST1* methylation in CTCs was confirmed by a multivariate analysis with each CTC individual marker in a Cox Regression model including established parameters like CTC count which is proven to be correlated with worse prognosis and Gleason score. Markowski et al. have shown that combination of CTC>5 and AR-V7(+) resulted to a better clinical management of mCRPC patients [43].

Our results based on this prospective liquid biopsy study using identical blood draws clearly indicate a significantly higher positivity of gene expression and tumor DNA methylation markers in EpCAM-positive CTCs compared to plasma-derived exosomes. We report that in EpCAM-positive CTCs, *CK-19*, *PSMA*, *TWIST1* expression and *GSTP1* methylation are significantly correlated with worse OS, while in exosomes, *CK-8* expression and *GSTP1* and *RASSF1A* methylation status were significantly correlated with a lower OS. Specific clinical trials with a much larger number of mCRPC patients are needed for the clinical evaluation of tdEVs enumeration as was done before for CTCs.

## 5. Conclusions

Our direct comparison study of CTCs and exosomes at the gene expression and DNA methylation level, revealed for the first time a significantly higher positivity in EpCAM-positive CTCs compared to plasma-derived exosomes. We report that in EpCAM-positive CTCs, *CK-19*, *PSMA*, *TWIST1* expression and *GSTP1* methylation are significantly correlated with worse OS, while in exosomes, *CK-8* expression and *GSTP1* and *RASSF1A* methylation status were significantly correlated with a lower OS. Specific clinical trials with a much larger number of mCRPC patients are needed for the clinical evaluation of tdEVs enumeration as was done before for CTCs.

## Figures and Tables

**Table 1:** Multivariate analyses for OS of mCRPC patients (*n* = 62).

Covariates	Covariates Value	Multivariate Cox Regression Analysis		
		HR ^a^	95%CI ^b^	*p*-Value
**CTC count**	≥5 vs. <5	2.202	1.013–4.786	**0.046**
**Gleason score**	>8 vs. ≤8	1.230	0.616–2.456	0.558
*CK19*	Yes vs. No	2.609	1.176–5.790	**0.018**
**CTC count**	≥5 vs. <5	1.957	0.885–4.324	0.097
**Gleason score**	>8 vs. ≤8	1.025	0.500–2.101	0.946
*PSMA*	Yes vs. No	3.242	1.536–6.846	**0.002**
**CTC count**	≥5 vs. <5	2.244	1.024–4.921	**0.044**
**Gleason score**	>8 vs. ≤8	1.610	0.774–3.350	0.202
*TWIST1*	Yes vs. No	4.421	1.744–11.209	**0.002**
**CTC count**	≥5 vs. <5	2.024	0.906–4.518	0.085
**Gleason score**	>8 vs. ≤8	1.275	0.641–2.539	0.489
*GSTP1*	Yes vs. No	3.111	1.163–8.321	**0.024**
**CTC count**	≥5 vs. <5	1.937	0.834–4.496	0.124
**Gleason score**	>8 vs. ≤8	1.234	0.572–2.663	0.592
*CK19*	Yes vs. No	1.418	0.553–3.634	0.467
*PSMA*	Yes vs. No	2.669	1.152–6.186	**0.022**
*TWIST1*	Yes vs. No	3.722	1.412–9.812	**0.008**
*GSTP1*	Yes vs. No	1.277	0.402–4.050	0.679

^a^ Hazard ratio, ^b^ Confidence interval of the estimated HR. Bold values denote statistical significance at *p* < 0.05.

## Data Availability

The data presented in this study are available on request from the corresponding author. The data are not publicly available due to ethical restrictions.

## Supplementary Materials

The following are available online at https://www.mdpi.com/2072-6694/13/4/780/s1, Figure S1: % Positivity of gene expression and DNA methylation markers in EpCAM positive CTCs in comparison to plasma-derived exosomes, Table S1: Clinical characteristics of mCRPC patients, Table S2: Direct comparison study of gene expression and DNA methylation markers in CTCs and exosomes derived from identical blood draws in mCRPC patients.
