# Supplementary material for: Prognostic Significance of Gene Expression and DNA Methylation Markers in Circulating Tumor Cells and Paired Plasma Derived Exosomes in Metastatic Castration Resistant Prostate Cancer
Source: Cancers (Basel). 2021 Feb 13;13(4):780. doi: 10.3390/cancers13040780 (PMC7918693; doi:10.3390/cancers13040780)
Supplement: Supplementary file 1 [file cancers-13-00780-s001.pdf]

Supplementary Materials

Prognostic Significance of Gene Expression and DNA Methylation Markers in Circulating Tumor Cells and Paired Plasma Derived Exosomes in Metastatic Castration Resistant Prostate Cancer

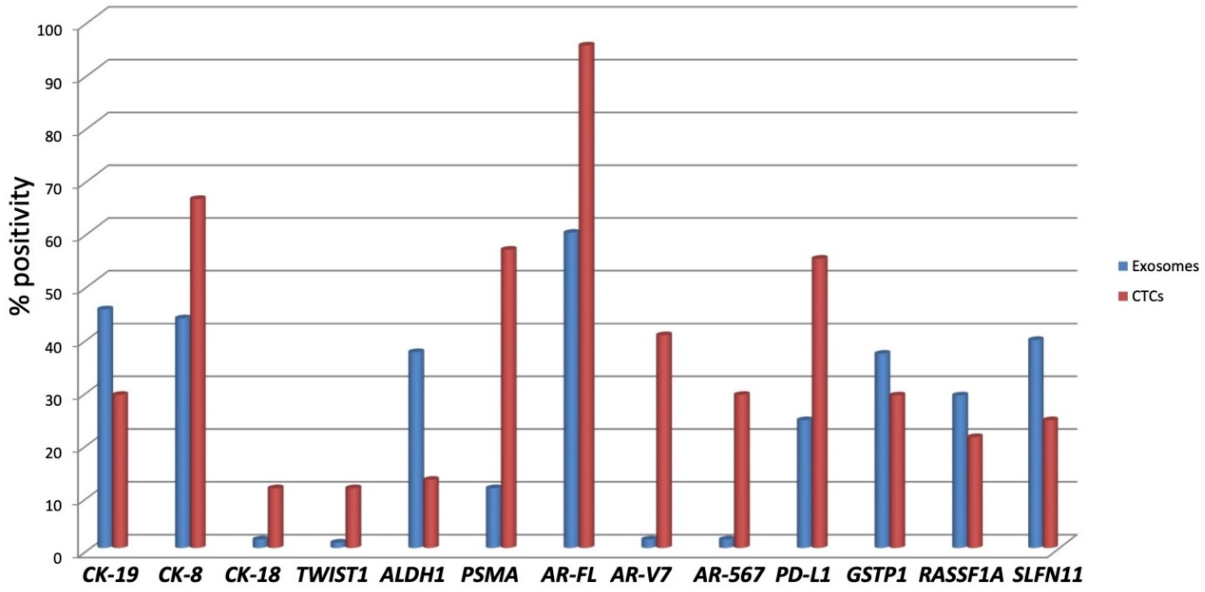

Figure S1. % Positivity of gene expression and DNA methylation markers in EpCAM positive CTCs in comparison to plasma-derived exosomes

Table S1. Clinical characteristics of mCRPC patients (*n* = 62)

**Variable**	**mCRPC patients (*n* = 62)**	
**Age**	<74	24(38.7%)
	≥74	24(38.7%)
	Unknown	14 (22.6%)
**Gleason score**	<8	16(25.8%)
	≥8	36(58.1%)
	Unknown	10(16.1%)
**PSA**	<50	26(41.9%)
	≥50	24(38.7%)
	Unknown	12(19.4%)
**Therapy**	Chemo	22(35.5%)
	NHA	20(32.3%)
	Unknown	10(16.1%)

Table S2. Direct comparison study of gene expression and DNA methylation markers in CTCs and exosomes derived from identical blood draws in mCRPC patients.

**Exosomes**	**CTCs**	**Concordance (*p*, Fisher's Exact test)**
**Gene expresion markers (RT-qPCR)**		
CK-19	***CK-19*** Negative Positive	
Negative	29 5	Concordance: 42/62, 68% (*p* = 0.011)
Positive	15 13	
CK-8	***CK-8*** Negative Positive	
Negative	14 20	Concordance: 35/62, 57% (*p* = 0.281)
Positive	7 21	
CK-18	***CK-18*** Negative Positive	
Negative	55 6	Concordance: 56/62, 90% (*p* = 0.113)
Positive	0 1	
TWIST1	***TWIST1*** Negative Positive	
Negative	52 5	Concordance: 54/62, 87% (*p* = 0.093)
Positive	3 2	

ALDH1	*ALDH1*<br>Negative Positive	
Negative	35 4	Concordance: 39/62, 63% ($p = 0.454$)
Positive	19 4	
PSMA	***PSMA***<br>Negative Positive	
Negative	26 29	Concordance: 32/62, 52% ($p = 0.126$)
Positive	1 6	
AR-FL	***AR-FL***<br>Negative Positive	
Negative	1 25	Concordance:35/62, 57% ($p = 1$)
Positive	2 34	
AR-V7	***AR-V7***<br>Negative Positive	
Negative	37 24	Concordance: 38/62, 61% (p= 0.403)
Positive	0 1	
AR-567	***AR-567***<br>Negative Positive	
Negative	43 18	Concordance: 43/62, 69%) ($p = 1$)
Positive	1 0	
PD-L1	***PD-L1***<br>Negative Positive	
Negative	24 23	Concordance: 35/62, 57% ($p = 0.139$)
Positive	4 11	
**DNA Methylation markers (MSP)**		
GSTP1	***GSTP1***<br>Unmethylated Methylated	
Unmethylated	21 3	Concordance: 28/38, 74% ($p = 0.021$)
Methylated	7 7	
RASSF1A	***RASSF1A***<br>Unmethylated Methylated	
Unmethylated	25 3	Concordance:30/38, 79% ($p = 0.019$)
Methylated	5 5	
SLFN11	***SLFN11***<br>Unmethylated Methylated	
Unmethylated	18 5	Concordance:25/38, 66% ($p = 0.157$)
Methylated	8 7	
